# Genomic Insights into the Spread of Vaccinia Virus Strain Cantagalo to Rural Regions of Northeastern Brazil

**DOI:** 10.3390/v18060629

**Published:** 2026-05-30

**Authors:** Maria Júlia Cadrieskt-Ribeiro, Matheus Nobrega Luques, Samuel Hir, Pedro Lucas O. Correia, Régis Linhares Oliveira, Carolina Maciel Neves, Keilla Maria P. Silva, Mayara Matias O. M. da Costa, Diego Arruda Falcão, Luciana Bahiense da Costa, Arabela Leal S. Mello, Jussara Lagos O. Silveira, Clarissa R. Damaso

**Affiliations:** 1 Instituto de Biofísica Carlos Chagas Filho, Universidade Federal do Rio de Janeiro, Rio de Janeiro 21941-902, RJ, Brazil; mjuliacribeiro@biof.ufrj.br (M.J.C.-R.); matheus.luques@biof.ufrj.br (M.N.L.); samuelhir@biof.ufrj.br (S.H.); pedrolucas@biof.ufrj.br (P.L.O.C.); regis.linhares@biof.ufrj.br (R.L.O.); carolinamaciel@biof.ufrj.br (C.M.N.); 2 Laboratório Central de Saúde Pública Dr. Milton Bezerra Sobral, Secretaria Estadual de Saúde, Recife 50050-250, PE, Brazil; keillampsilva@gmail.com (K.M.P.S.); mayaramatiasoliveira@gmail.com (M.M.O.M.d.C.); diego_arruda2006@hotmail.com (D.A.F.); 3 Diretoria de Vigilância Epidemiológica da Secretaria de Saúde do Estado da Bahia, Secretaria Estadual de Saúde, Salvador 41745-002, BA, Brazil; lubahiense@hotmail.com; 4 Laboratório Central de Saúde Pública Professor Gonçalo Moniz, Secretaria Estadual de Saúde, Salvador 41745-900, BA, Brazil; arabelaleal@gmail.com (A.L.S.M.); jussara.silveira@saude.ba.gov.br (J.L.O.S.); 5 Núcleo de Enfrentamento e Estudos de Doenças Infecciosas Emergentes e Reemergentes (NEEDIER), Universidade Federal do Rio de Janeiro, Rio de Janeiro 21941-902, RJ, Brazil

**Keywords:** orthopoxvirus, vaccinia virus, poxvirus, recombination, genetic diversity

## Abstract

Vaccinia virus strain Cantagalo (CTGV) causes a pustular disease in dairy cows and milkers in Brazil. Outbreaks in several states have been frequently reported, but the full genome sequence and genomic analysis of isolates from the Northeast region have never been described. Here, we report CTGV outbreaks in two Northeastern states, affecting milkers, lactating cows, and suckling calves. The farms were located in the main dairy belt of Pernambuco, in the Borborema Plateau, and in a rural region of Bahia. Of the 12 samples that tested positive for CTGV, five had their genomes fully sequenced. They cluster with CTGV isolates from Goiás (Midwest region, 2022) and São Paulo (Southeast region, 2023) but diverge from isolates from the Southeast in the early 2000s. Two clinical isolates have accumulated greater genetic variability and segregate separately from the other three isolates from the Northeast, showing evidence of potential recombination events with the FAI-01 isolate from the Midwest region (2022). We also detected *Parapoxvirus* and CTGV coinfection in some animals. These findings likely suggest different episodes of virus introduction in these states. The spread of CTGV raises concerns about the potential impact on local economic activities and underscores the importance of avoiding raw milk consumption.

## 1. Introduction

Vaccinia virus (VACV) is taxonomically assigned to the species *Orthopoxvirus vaccinia* (genus *Orthopoxvirus*, family *Poxviridae*), and some strains have long been used to manufacture smallpox and, more recently, mpox vaccines [1,2]. The occurrence of natural VACV infections is restricted to a few countries, where field strains are the etiologic agent of vesiculopustular disease in buffaloes and dairy cows, and milkers, usually referred to as bovine vaccinia [3]. Although a rare phenomenon in the world, this zoonotic disease is endemic in rural areas of Brazil, Colombia, and India [4,5,6,7,8,9]. It is characterized by fever, axillary lymphadenopathy, vesiculopustular lesions on the hands and arms of milkers and on the teats and udder of dairy cattle, sometimes accompanied by secondary bacterial infection [10].

VACV infection is generally self-limiting and resolves in approximately three weeks, but complications may occasionally occur [11,12]. Milkers acquire the infection occupationally after contact with the injured teats and udders of lactating cattle, but, in addition to skin contact, virus transmission can also occur through the consumption of raw milk [4,13]. Milk production must be interrupted for several weeks in infected animals, and secondary mastitis is a frequent complication, causing important economic losses, especially on manual milking farms [14].

VACV strain Cantagalo (CTGV) was first detected in dairy cattle and milkers of a farm in the state of Rio de Janeiro, Brazil, in 1999 [4,15]. Since then, CTGV and VACV isolates closely related to CTGV have been detected in different states, leading to outbreaks in several regions of Brazil [7,8,15,16,17,18,19,20]. Despite this widespread and frequent occurrence over the past 25 years, knowledge of the genetic diversity of VACV field isolates remains scarce, with only 13 genome sequences available in public databases.

None of these 13 genomes correspond to VACV isolates from Northeast Brazil, although VACV outbreaks were reported in Northeastern states in the 2010s, and the isolates were phylogenetically related to CTGV based on the sequence of some genes [18,19,21,22]. However, information is lacking regarding the complete genome sequence of VACV isolates circulating in this region, as well as their genomic structure and detailed genetic relationships.

In this work, we describe outbreaks of CTGV infection in the states of Pernambuco and Bahia, between 2023 and 2024. We also describe and analyze the full-length genomes of five clinical isolates and investigate their genetic relationships. Genomic analysis of VACV isolates is relevant for genomic surveillance and for investigating evolutionary relationships and potential impacts on the genetic makeup of circulating VACV, which could lead to future changes in virulence.

## 2. Materials and Methods

### 2.1. Samples, Virus Isolation, and Molecular Diagnosis

**Scheme 1:**
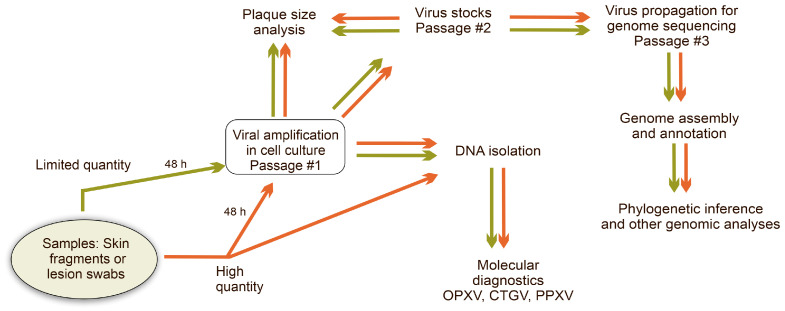
Experimental flowchart. Samples with sufficient material proceeded directly to DNA isolation, whereas limited samples required initial viral amplification. All subsequent steps of the workflow remained uniform across both groups.

Samples of skin fragments and lesion swabs with restricted amounts of material were initially macerated in Puck’s saline solution, and the supernatant was used to infect BSC-40 cells (*Chlorocebus aethiops* kidney cells) for viral amplification (Scheme 1) [17]. After 48 h, when 100% cytopathic effect was achieved, cells were harvested for virus stock, stored at −80 °C, and part was used for DNA isolation. Samples with a larger amount of material were macerated in sterile 0.9% saline solution, and after clarification, part of the supernatant was used to infect cells to obtain virus stocks, as described above, and part was used for DNA isolation (Scheme 1). Virus stocks were subsequently used for determining virus titers via plaque assay in BSC-40 cells [4] and visualization and inference of viral plaque phenotype in BSC-40 and HeLa cells (human cervical adenocarcinoma cell line) [7]. DNA was extracted using cold Nuclei Lysis Buffer added to the harvested cells or to the clarified material from the macerated clinical samples. The procedure followed the manufacturer’s instructions for the Wizard^®^ Genomic Purification Kit (Promega, Madison, WI, USA), as described [7,17].

DNA was used in real-time PCR assays targeting the *Orthopoxvirus* (OPXV) *F4L* gene, as described [7,23]. Reactions with Ct values above 37 were considered negative, based on a dilution curve of purified VACV DNA. A conventional PCR/nested-PCR approach was applied to detect CTGV specifically. As described, the OPXV hemagglutinin gene was first amplified using EACP1/910rev set of primers [8,16]. Amplicons were submitted to a subsequent nested-PCR assay, using primers that anneal to a CTGV-specific region within the hemagglutinin amplicon [17]. The presence of the virus of the genus *Parapoxvirus* in samples was evaluated using real-time PCR assays targeting the *J6R* gene [24]. Based on dilution curves using *Parapoxvirus* DNA, samples with Ct values below 35 were considered positive.

### 2.2. VACV Whole Genome Sequencing

Virus stocks (passage #2) from selected isolates were used to infect BSC-40 cells for 48 h at 37 °C. Cell monolayers were then harvested, disrupted in Dounce homogenizers (Bellco, Vineland, NJ, USA), and post-nuclear extracts were centrifuged at 18,000× *g* for 60 min at 4 °C to sediment cytoplasmic virosomes. DNA from the viral factories was extracted, using the Wizard^®^ Genomic Purification Kit (Promega, Madison, WI, USA), as described above [15]. DNA libraries were constructed using the DNAprep kit (Illumina, San Diego, CA, USA), following the manufacturer’s instructions, quantified by Quantus (Promega), and used for sequencing in the Illumina NextSeq 1000 system (Illumina) [7]. After passing quality control (FastQC v012.1 and MultiQC v1.29 tools), cellular reads were filtered out in Bowtie v2.4.4 [25], and viral reads were de novo assembled in SPAdes v3.15.4. [26]. Geneious v2023.0.1 was used to correctly assemble the Inverted Terminal Repeats (ITRs) and subsequently to map the reads against the final assembled genome (Geneious mapper using default parameters) in order to assess the depth of coverage and check for inconsistencies in the assembly. The assembled genomes were annotated using GATU and CLC Main Workbench v23.01 [7,15], and submitted to the Genbank public database with the following accession numbers: PX423480 (BC-02), PX423481 (BC-04), PX423482 (LO-01), PX423483 (TE-09), and PX423484 (IBI-05).

For partial sequencing of the *Parapoxvirus B2L* gene, conventional PCR/nested PCR targeting the *B2L* gene was performed, as described [27], and the amplicons were purified and Sanger-sequenced, as described [16]. Contigs were assembled using Geneious v2023.0.1 and submitted to the GenBank public database with the accession number PZ157926.

### 2.3. VACV Whole Genome Analyses

For phylogenetic analysis, the five genomes sequenced in this study were aligned with 57 other OPXV genomes using Mafft v7 server with default parameters [28] and after visual inspection and gap removal, the conserved region was trimmed (approximately 95,000 bp) and used as input in IQ-TREE multicore 1.6.12 [29] to generate a Maximum Likelihood tree, opting for Kimura-3-p model with empirical base frequencies, 3-category free rate model, and 1000 bootstraps. The resulting tree was visualized in Mega 11. Accession numbers of the OPXV genomes used in all genetic analyses are listed as Supplementary Material (Table S1).

Full-length CTGV genomes were aligned using the Mafft server with default parameters [15]. Alignments were used to infer scores of nucleotide identity using CLC Main Workbench v23.01 [7,15]. Graphical presentation of single-nucleotide polymorphisms (SNPs), insertions and deletions (INDELs) distributed along the CTGV genomes was implemented in Base-by-base v3 [30]. Gaps and identical residues in the alignments were extracted using Mega 11. The subsequent nexus file was implemented in Population Analysis with Reticulate Trees (PopART v1.7.1), with epsilon = 0 (default parameters) to generate a Median Joining Network [31].

To detect pairwise synteny blocks in CTGV genomes, gff3 and fasta files were generated from annotated genomes of different CTGV using CLC Main Workbench v23.01 and used as input in MCScanX from TBtools-II version 2.441 [32], opting for an e-value of 1 × 10^−10^ and a blast match number of 5. The output files were manually checked to ensure correct ortholog mapping. The layout files, linked genes, and position data of synteny gene pairs from all CTGV synteny gene blocks were merged into three tabular files using Excel and used as input to generate a synteny plot using the Multiple Synteny Plot tool of TBtools-II version 2.441.

To generate similarity plots, sequence alignment datasets were fed into Simplot v.3.5.1, opting for kimura-2p and Gapstrip on. The Bootscan tool, implemented in Simplot v.3.5.1, was used to detect potential recombination regions, using the same parameters described above and the neighbor-joining tree model [33]. Values of window size and step size are specified in the figure legends.

## 3. Results

### 3.1. Case Description and Virus Detection

**Figure 1:**
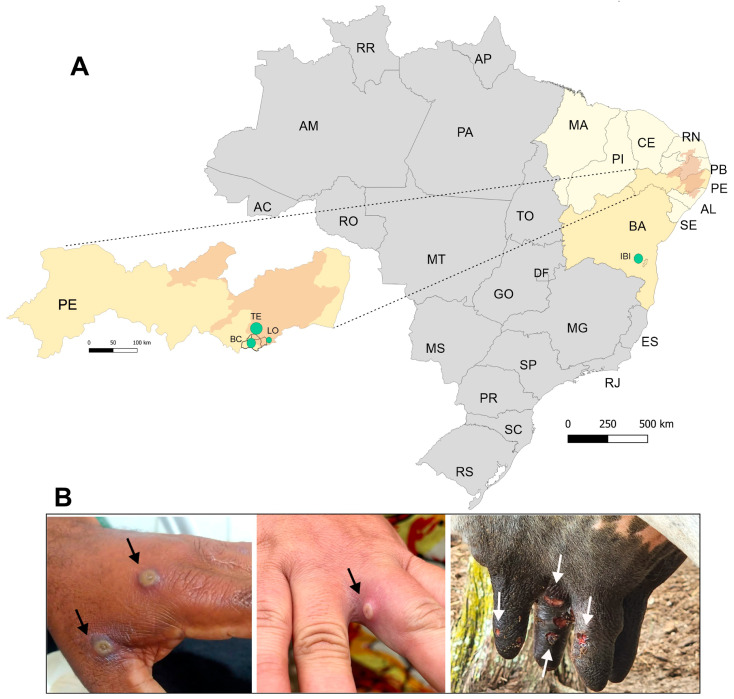
Detection of vaccinia virus strain Cantagalo in ecotourism and rural areas of Northeastern Brazil. (**A**) Map of Brazil highlighting the Northeast region (light beige), the states of Pernambuco (PE) and Bahia (BA) shown in yellow, all other states shown in gray (QGIS: 3.34.11-Prizren, https://qgis.org/). The size of the circles corresponds to the number of CTGV cases detected in each municipality (green circles), which are delimited in the map by thin lines. The Borborema Plateau, which extends across four states of the Northeast region, is shown in brown. (**B**) The representative lesions on the hands of milkers and on the teats of a lactating cow that were positively diagnosed with CTGV infection. The arrows indicate clinical signs. Photographs by LACEN-PE and LACEN-BA.

From November 2023 to February 2024, the Central Laboratory of Public Health of the state of Pernambuco (LACEN-PE) reported 15 cases of pustular disease suspected of bovine vaccinia. Patients were rural workers from farms located in three neighboring municipalities, 17 to 30 km apart: Terezinha, Lagoa do Ouro and Bom Conselho. These municipalities are located in the Agreste mesoregion, on the Borborema Plateau. This mesoregion is the main dairy belt of Pernambuco, and Bom Conselho is the sixth-largest milk producer in the state [34] (Table 1, Figure 1A).

Rural workers presented isolated pustular lesions in their hands and arms, and dairy cows on the affected farms also had pustular lesions in the teats (Figure 1B). Malaise, local edema, fever, asthenia, and muscle ache were also reported. In July 2024, an outbreak of pustular disease with similar characteristics was reported by the Central Laboratory of Public Health of the state of Bahia (LACEN-BA) on a dairy farm in Ibicuí, southern Bahia, and subsequently, in December 2025, on a dairy farm in Ilhéus, 134 km from Ibicuí (Table 1, Figure 1A). Both municipalities are located in a farming region with thriving rural and tourist activities.

Skin fragments and/or lesion swabs were collected from 15 patients in Pernambuco and from 1 patient, 4 lactating cows, and 1 suckling calf in Bahia for molecular diagnosis. Four swab-derived DNA samples, TE-02, TE-03, IBI-04, and ILH-01, were negative for OPXV (Ct ≥ 37.1), but the other 17 samples were positive for OPXV with Ct ≤ 37 (Table 1).

To further investigate whether the infection was caused by CTGV, a conventional, CTGV-specific nested PCR assay was implemented, as described in Methods [17]. Of the 17 OPXV positive samples, 12 were positive for CTGV based on the presence of the 714-bp band in agarose gels (Table 1). The 5 samples that were negative for CTGV had Cts ≥ 32 in qPCR assays for OPXV, indicating low viral loads in the samples and, therefore, lower chances of virus isolation in cell culture or detection of amplicon bands after conventional PCR (Table 1). Still, the results confirmed CTGV circulation in both northeastern states.

We also tested all samples, except three with insufficient amounts of DNA, for the presence of *Parapoxvirus* (PPXV) using a qPCR assay targeting the *J6R* gene [24]. PPXV can cause a disease in dairy cows and humans that is clinically indistinguishable from CTGV infection and often occurs after or overlaps with CTGV outbreaks [17], as they share similar transmission routes [35]. Two CTGV negative samples from Bahia, ILH-01 and IBI-04, were positive for PPXV (Ct < 35) and two CTGV positive samples, IBI-03 and IBI-05, showed co-infection with PPXV, although with a low viral load (Table 1). All samples from Pernambuco were negative for PPXV. Insufficient amounts of DNA prevented us from identifying the PPXV species, except in the sample IBI-03. The *B2L* gene amplicon (512 bp partial sequence) of IBI-03 was Sanger sequenced, followed by a Blastn search of the resulting sequence, which indicated the first 84 results as pseudocowpox virus, with a query coverage of 89–96% and an identity of 95.14–97.76%.

### 3.2. Genotypic Diversity of the CTGV Isolates from Northeast Brazil

To investigate the genotypic diversity of the CTGV isolates circulating in both states, we selected 5 clinical isolates (TE-09, BC-02, BC-04, LO-01, and IBI-05) for whole genome sequencing using Illumina NextSeq 1000. Sequencing statistics are described in Table 1. The 5 clinical isolates had genome sizes similar to those of other previously sequenced CTGV isolates, ranging from 186,175 kb to 187,173 kb.

**Figure 2:**
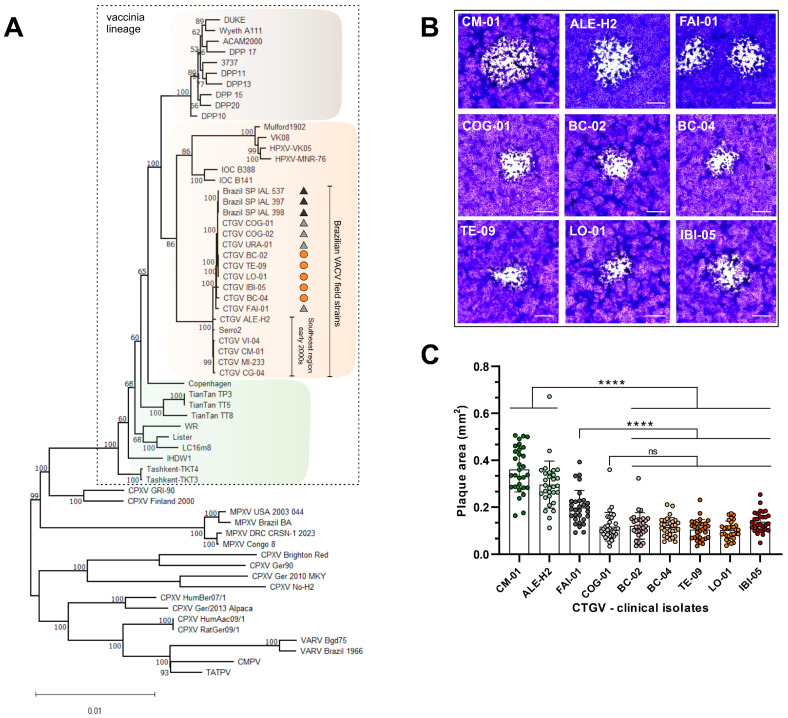
Phylogenetic analysis and viral plaque phenotype of CTGV isolates from Northeastern Brazil. (**A**) Maximum likelihood phylogenetic tree of the conserved region (approximately 95,000 bp) of 62 orthopoxvirus genomes, opting for the Kimura-3-p model with empirical base frequencies, 3-category free rate model, and 1000 bootstraps. The three VACV clades are included in the dotted box (vaccinia lineage) and are shown in brown (clade I), orange (clade II), and green (clade III). Orange circles: CTGV isolates from Pernambuco (BC-02, BC-04, TE-09, and LO-01) and Bahia (IBI-05) sequenced in this work. Gray and black triangles: CTGV genomes from Goiás, 2022 and São Paulo, 2023, respectively. CPXV: cowpox virus, MPXV: monkeypox virus, CMPV: camelpox virus, TATPV: taterapox virus. Bootstrap values > 50% are shown next to branch nodes. The scale bar indicates the number of substitutions per site. (**B**) BSC-40 cells infected with the indicated CTGV isolates were fixed and stained with 10% formaldehyde/0.1% crystal violet. Representative viral plaques were photographed, scale bars: 250 µm. (**C**) the area of thirty random plaques was determined using Image J v1.54. CM-01: CTGV isolate from RJ, 1999. ALE-H2: isolate from Espírito Santo, 2006; COG-1: CTGV isolate from GO, 2022. FAI-01: CTGV isolate from GO, 2022. Mean ± SD; asterisk: *p* < 0.0001 (One-way ANOVA followed by Bartlett’s test); ns: not significant.

Phylogenetic inference shows that, as expected, the 5 isolates clustered within clade II of the vaccinia lineage, along with other previously sequenced CTGV isolates (Figure 2A). However, these 5 isolates mapped closer to CTGV isolates from 2022 and 2023 from Goiás (GO, Central-West region) and São Paulo (SP, Southeast region), respectively, than to isolates from the Southeast region from the early 2000s (Figure 2A). A more defined topology of the CTGV subtree, highlighting these relationships, is shown in Figure S1. Identity scores for the whole genome sequences confirm these observations and reveal 99.29 to 99.82% identity with isolates from 2022 to 2023 and lower identity values (96.42–98.99%) with clinical isolates from the early 2000s, including the first CTGV isolate, CM-01.

Interestingly, all 5 clinical isolates sequenced in this work generated small viral plaques in BSC-40 cell monolayers, similar to those of clinical isolates from the state of Goiás (2022), such as COG-01 and even smaller than the plaques generated by the FAI-01 isolate, also from Goiás (2022). However, plaques generated by the 5 CTGV isolates from the Northeast region were, on average, 2.7 times smaller than those produced by clinical isolates from the early 2000s of the states of Rio de Janeiro (CM-01, 1999) and Espírito Santo (ALE-H2, 2006). These latter strains generate significantly larger viral plaques (Figure 2B,C and Figure S2), which is usually related to an improved ability to spread in cell culture [4]. Similar results were obtained when plaques were generated in HeLa cells (Figure S3).

**Figure 3:**
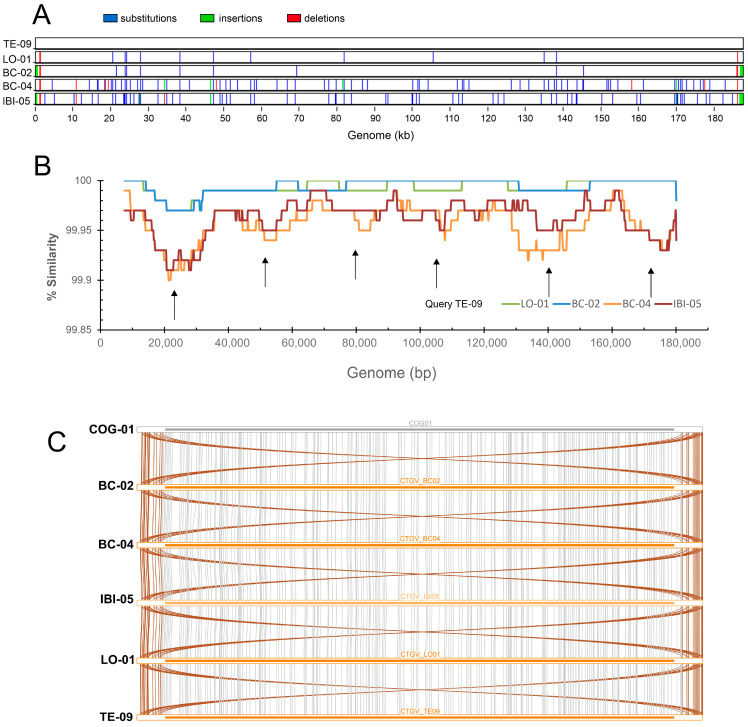
Genome analysis of five CTGV isolates from Pernambuco and Bahia. (**A**) Graphical distribution of SNPs and INDELs across the genomes of the isolates TE-09, LO-01, BC-02, and BC-04 from Pernambuco, and IBI-05 from Bahia. Isolate TE-09 was selected as the reference genome. (**B**) Similarity plot: the alignment was used as input for Simplot, opting for TE-09 as query, window size of 15,000, and window step of 200. Arrows indicate regions of greater divergence from the query. (**C**) Pairwise detection of synteny blocks in the 5 CTGV genomes sequenced in this study and COG-01 from Goiás (2022), using MCScanX from TBtools-II. A synteny plot was generated using the tool Multiple Synteny Plot. Synteny gene pairs are linked by gray lines, except for genes in the ITRs, which are linked by brown lines.

Although the 5 isolates from the Northeast region exhibit similar plaque phenotypes, their genomes accumulate genetic differences among themselves, as shown by the graphic distribution of SNPs and INDELs along the whole genomes (Figure 3A). Isolates TE-09, LO-01, and BC-02 from Pernambuco are more similar to each other, showing few genetic variations, while BC-04 and IBI-05, from Pernambuco and Bahia, respectively, are more divergent, with a prevalence of SNPs over putative INDELs. Isolates BC-04 and IBI-05 share some SNPs and INDELs, but most are in different genome positions, although they tend to accumulate within the same regions of the genome (Figure 3A).

The genome-wide profile of similarity was scored and corroborated the base-by-base findings (Figure 3B). Using isolate TE-09 from Pernambuco as a reference genome, we can observe that both isolates BC-04 and IBI-05 have lower similarity across the whole genome of TE-09 when compared with LO-01 and BC-02. As also observed in Figure 3A, genetic variation in the genomes of BC-04 and IBI-05, which confer less similarity to TE-09, LO-01, and BC-02, concentrates in the same genomic regions (Figure 3B, arrows). However, these regions of divergence do not reflect alterations in syntenic gene blocks within the BC-04 and IBI-05 genomes. A genome-wide inference of collinear chains of homologs was performed using the 2022 isolate COG-01 as a reference genome. As shown in Figure 3C, all paired blocks of genes (gray lines) are in synteny across the whole genomes and genes located in one Inverted Terminal Repeat (ITR) region are duplicated in the other ITR (Figure 3C, brown lines).

**Figure 4:**
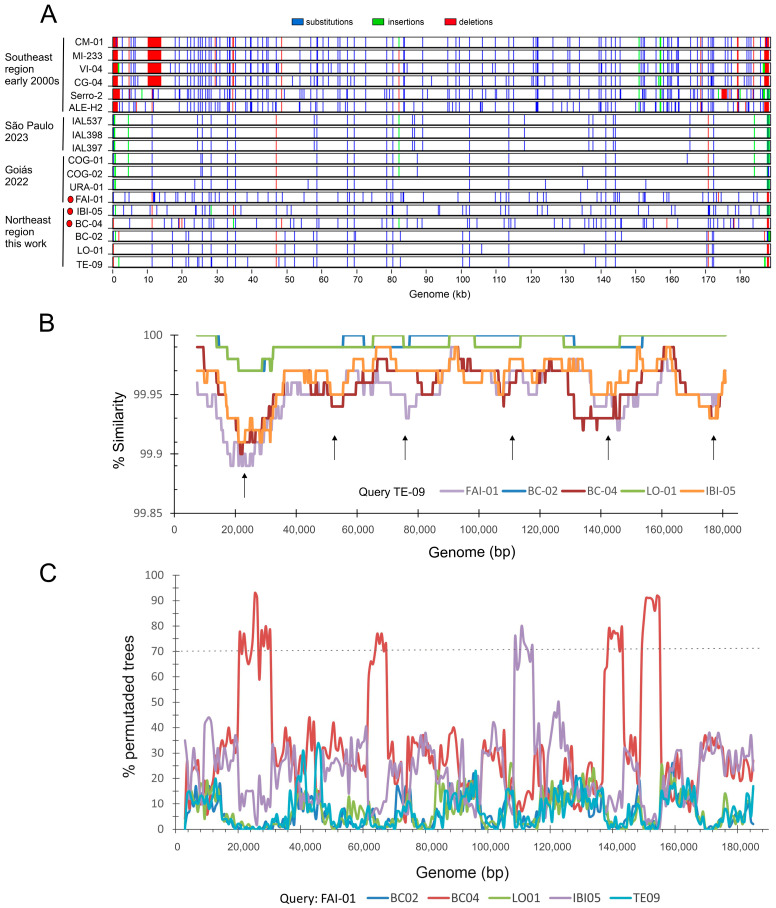
Genome-wide analysis of CTGV isolates. (**A**) Eighteen CTGV genomes were aligned and submitted to a genome-wide screen for SNPs and INDELs, opting for comparison against a consensus sequence. Red circles indicate the CTGV genomes of FAI-01, IBI-05, and BC-04. (**B**) Similarity plot: the alignment of the 5 CTGV genomes sequenced in this study, and FAI-01 (Goiás, 2022) was used as input for Simplot, opting for TE-09 as query, window size of 15,000 and window step of 200. Arrows indicate regions of greater divergence from the query. (**C**) Bootscan plot: the alignment shown in (**B**) was fed into the Bootscan tool, choosing FAI-01 as query, window size of 6000 and window step of 700. Evidence of recombination was considered when >70% of permutated trees (dotted lines) supported the analysis.

A genome-wide comparison of all CTGV sequences was implemented to investigate the distribution of SNPs and putative INDELs across the 5 genomes sequenced in this work when compared to previously sequenced CTGV genomes. Figure 4A shows a striking difference between genomes from outbreaks reported in the early 2000s and genomes from outbreaks that occurred after 2022. We also observe that the isolate FAI-01 from the state of Goiás (GO, 2022) substantially diverges from other CTGV isolates obtained from simultaneous outbreaks in 2022 (COG-01, COG-02, and URA-01) [7]. Although the variation profile is not identical to BC-04 and IBI-05, these 3 isolates share the same regions of divergence (Figure 4B, arrows). A Bootscan recombination analysis, using the CTGV isolate FAI-01 as a query, suggests that both BC-04 and IBI-05 share potential recombination regions with FAI-01, with four of these regions occurring between BC-04 and FAI-01 (Figure 4C). These potential recombination segments are located in regions of similarity between FAI-01 and BC-04 or IBI-05, shown in Figure 4B, suggesting a close relationship between these three CTGV isolates that warrants further investigation.

**Figure 5:**
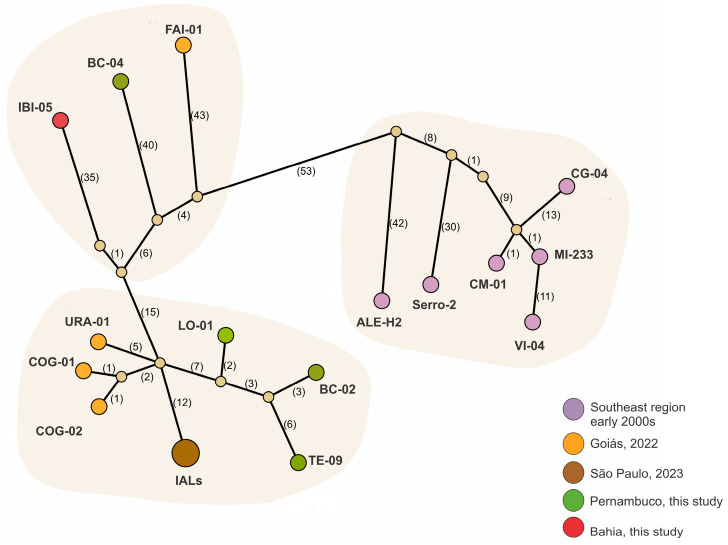
Median-Joining Network Analysis of CTGV isolates. The alignment of 18 genomes of CTGV isolates and related VACVs, containing only sites with sequence variations, was used as input for the PopART software to generate a Median-Joining Network using default parameters. Edge lengths are not necessarily proportional to the number of SNPs, which are indicated in parentheses next to each edge. The small beige nodes (median vectors) indicate unsampled or extinct common ancestors. Each color-coded vertex at the end of the edges corresponds to a virus isolate.

To better estimate the genetic distance and most likely connections between these genomes, we constructed a median-joining network (MJN) that highlights the number of substitutions separating the viral genomes from unsampled or unobserved sequences reconstructed in the network (Figure 5). We detected three subgroups of genomes. The isolates TE-09, LO-01, and BC-02 clustered with isolates COG-01, COG-02, and URA-01 from the state of Goiás (GO, 2022) and the three identical isolates from São Paulo (SP, 2023). These isolates diverge by a few SNPs and a few unsampled or extinct common ancestors. On the other hand, isolates BC-04 and IBI-05 mapped to another group together with the isolate FAI-01 from Goiás (2022), although with more divergence among each other than the first group. A third group contained all CTGV genomes from the Southeast region dating back to the early 2000s (Figure 5).

Table S2 lists the alterations in CDS regions unique to the CTGV isolates described in this study. As expected, strains BC-04 and IBI-05 exhibit more alterations than the others, which is consistent with the genomic analysis of SNPs and INDELs performed in this study. Most of the changes were silent, and no changes were detected in genes already known to affect VACV plaque size, such as *B5R*, *F5L*, *F11L*, *F12L*, *F13L*, *A34R* and *A36R* [36], except for a silent change in *A33R* of IBI-05. However, the *C2L* ortholog in isolate BC-04 exhibits two deletions of 1 and 2 nucleotides, which cause frameshift mutations and the introduction of premature stop codons. Deletions were confirmed by read mapping, which showed no inconsistencies. The deletion closest to the 5′ end of the gene leads to the premature termination of the C2 protein, which is predicted to have 123 amino acids, instead of the 512 amino acids of the other intact *C2L* orthologs. Another frameshift mutation is detected in the *K5L* ortholog of the IBI-05 isolate. However, the K5 is truncated in all CTGV isolates sequenced to date, as well as in several VACV strains. Furthermore, the BC-04 and IBI-05 isolates exhibit several missense alterations in essential genes, such as *A22R*, *J6R*, and *H2R*, among others, and all isolates present missense changes in genes involved in immunomodulation. The impact of these amino acid substitutions on protein functions needs further detailed evaluation.

## 4. Conclusions

Our work demonstrates the spread of the VACV strain Cantagalo to Northeast Brazil and highlights the accumulation of genetic variability among clinical isolates. Although genome sequencing was not performed directly from clinical samples, viral DNA was obtained after a low number of virus passages (passage #3) in cell culture to minimize the introduction of artificial alterations due to in vitro replication cycles.

The CTGV isolates from the Northeast region are closer to those from the Central-West region (2022) and São Paulo (2023), and more divergent from the first CTGV isolates from the 2000s [4,12,17]. However, one CTGV isolate from Pernambuco (BC-04) and one from Bahia (IBI-05), although close to the 2022 and 2023 isolates, segregate separately from the other three isolates from the Northeast and form another genetic group along with the CTGV isolate FAI-01, also isolated in the Central-West region in 2022. Evidence of recombination events in these genomes reinforces their relatedness and corroborates the hypothesis that the outbreaks described in this study likely resulted from different episodes of virus introduction in Pernambuco and Bahia. Interestingly, BC-02 and BC-04 isolates were collected in the municipality of Bom Conselho, nearly one month apart from each other, but on different farms. These results indicate that CTGV was likely already circulating in Northeast Brazil before 2023.

In this context, previous studies from the 2010s reported the detection of VACV isolates in the northeastern states of Maranhão, Pernambuco, and Bahia. These studies reported the partial nucleotide sequence of some genes, including the *A56R*, *A25L*, and *B5R* genes of two VACV isolates that clustered with CTGV in phylogenetic inferences [18,19,21,22]. The alignment of these partial sequences revealed some unique SNPs and INDELs, not present in the orthologs of the CTGV isolates described here, suggesting that the VACV isolates from the 2010s may not be the ones currently circulating, which supports the hypothesis that new VACV introductions have occurred over the years, as discussed above. However, only the complete genome sequencing of the isolates would allow us to confirm or rule out this hypothesis. Sequencing more genomes related to the outbreaks in both states and detailed genomic analysis are needed to unravel these questions.

As mentioned earlier, evidence of potential recombination events was detected between isolates BC-04 and IBI-05 and FAI-01 from Goiás (2022), and a detailed analysis of the recombined regions will be investigated in the future. However, as for the other isolates, no robust evidence of recombination was detected between the two isolates BC-04 and IBI-05 and the other 3 isolates described in this study. Poxviruses are well known for their improved ability to recombine as a mechanism of natural evolution [37]. Therefore, co-circulation of different orthopoxviruses in the same region should serve as a warning sign to search for possible recombinant viruses. Recently, this concern was raised when CTGV was detected in the rural municipality of Uruana, in Goiás, in 2022, where cases of monkeypox virus had been reported earlier in the same year. However, analysis of the genome sequences ruled out any potential recombination events [7]. Even so, human-to-human transmission of the monkeypox virus still occurs, as do outbreaks of CTGV, which reinforces the need to intensify genomic vigilance.

The unique alterations in the CTGV genomes from the Northeast region, located in CDS regions, did not initially reveal any major changes, as most of the changes were silent. Furthermore, inactivation of genes already known to affect VACV plaque size was not detected [36]. Interestingly, however, the C2 protein encoded by the BC-04 isolate is truncated. C2 is a BTB (Bric-a-brac, Tramtrack and Broad-complex)-Kelch protein that has been shown to be an inhibitor of NF-κB and to contribute to the formation of cellular projections [38,39]. All other CTGV isolates sequenced to date possess intact *C2L* orthologs, but not other VACV strains, such as MVA and IOC (some clones), in which *C2L* is inexistent [39] or the ORF is truncated [40]. Although C2 is truncated in BC-04, no alteration in cytopathic effect and cellular projections was observed compared to other isolates. However, studies that defined the role of C2 have generated mutants with a complete deletion of the gene [38,39]. BC-04 isolate, on the other hand, is predicted to produce a small fragment of the protein. Further research is needed to estimate the impact of truncated C2 on the BC-04 replicative cycle.

Isolates BC-04 and IBI-05 also exhibit several missense alterations in essential genes, such as *A22R*, which encodes Holliday junction resolvase, *J6R* (RPO147 subunit of RNA polymerase), and *F10L* (serine-threonine kinase 2). Furthermore, all isolates exhibited missense alterations in important virulence genes, such as *M2L* (NF-κB inhibitor), *B8R* (soluble interferon-II receptor-like protein), and *B19R* (secreted IFN-I receptor-like glycoprotein). However, it was not possible to determine whether or not these alterations affect protein function, since residues important for protein function have not yet been fully mapped for most genes. These alterations warrant further investigation.

Another interesting observation from this study was that *Parapoxvirus* (PPXV) was the etiological agent of the outbreak in Ilhéus, Bahia (2025), and not VACV. The most interesting finding was that two cows from the previous outbreak in Ibicuí, Bahia (2024), were co-infected with CTGV and *Parapoxvirus*, the latter being genetically identified as pseudocowpox virus. Detection of PPXV in dairy cattle in Brazil is not unusual, and coinfection with VACV has already been described [17,41,42,43].

Bahia and Pernambuco are the first and third-highest-ranked states in tourism in Northeastern Brazil [44]. Beyond the coastal route, ecotourism and rural tourism are on the rise, attracting many travelers recently, driven primarily by the post-pandemic preference for local and outdoor experiences. The municipalities with CTGV cases in Pernambuco are located in the Borborema Plateau, which is part of the Brazilian Highlands and extends across four states of the Northeast region. The Plateau is one of the emerging regions for ecotourism and geotourism, combining diverse ecosystems, from the Caatinga biome to the Atlantic Forest, and unique reliefs and important geomorphological sites [45,46]. In addition to this potential, the municipalities of Terezinha, Bom Conselho, and Lagoa do Ouro are located within the Agreste Mesoregion, home to the state’s main dairy belt [34]. Bahia, in turn, has several farms and community initiatives that have been successfully investing in rural tourism and day-use farming experiences, a growing practice on various farms in Brazil, including the farm in Ibicuí where the CTGV cases were detected. The emergence of CTGV in this region raises economic concerns due to its impact on local ecotourism activities and the dairy trade.

Our work also serves as a warning to avoid the consumption of raw milk, as transmission of VACV has been shown to occur through this route [13]. It is worth noting that, in this study, one of the infected animals was a suckling calf with lesions in the oral cavity (clinical sample IBI-05). Consumption of raw milk is a common habit on small family farms and, despite warnings from various health regulatory agencies, the practice is growing among certain segments of the population, particularly those who advocate a more natural and unprocessed diet [47]. Raw milk can be a source of several pathogenic bacteria and viruses, such as H5N1, Rift Valley fever, tick-borne encephalitis, and CTGV, among others [48].

## Figures and Tables

**Table 1:** Clinical isolates from Pernambuco and Bahia, Brazil, were used in this study.

Municipality	Laboratory Designation	Isolate Origin	OPXV Ct Values	CTGV PCR	PPXV Ct Values	CTGV Genome Size (bp)	Mapped Reads/Total Reads ***	Genome Coverage
**Terezinha, PE**	TE-01	Human	32.58	N/D	N/D			
	TE-02	Human	N/D	N/D	N/D			
	TE-03	Human	N/D	N/D	N/D			
	TE-04	Human	33.36	N/D	N/D			
	TE-05	Human	23.27	+	N/D			
	TE-06	Human	36.18	N/D	N/D			
	TE-07	Human	34.04	N/D	N/D			
	TE-08	Human	33.87	N/D	N/D			
	TE-09	Human	14.2	+	N/D	186,391	198,462/ 5,994,246	329×
**Bom Conselho, PE**	BC-01	Human	12.25	+	I			
	BC-02	Human	13.05	+	N/D	186,855	90,493/ 4,727,392	144×
	BC-03	Human	12.55	+	I			
	BC-04	Human	14.14	+	N/D	186,195	184,446/ 4,583,624	290×
	BC-05	Human	14.09	+	I			
**Lagoa do Ouro, PE**	LO-01	Human	14.21	+	N/D	186,175	34,734/ 4,770,590	55×
**Ibicuí, BA**	IBI-01	Bovine	26.81	+	N/D			
	IBI-02	Bovine	29.91	+	N/D			
	IBI-03	Bovine	32.68	+	31.81 **			
	IBI-04	Bovine	N/D	N/D	34.87			
	IBI-05	Bovine *	14.12	+	32.77	187,173	42,899/ 4,536,868	68×
**Ilhéus, BA**	ILH-01	Human	N/D	N/D	22.58			

PE: state of Pernambuco; BA: state of Bahia. * Suckling calf. N/D: not detected. +: positivity in conventional PCR. I: insufficient samples. ** Positivity confirmed by conventional PCR for *Parapoxvirus* detection. *** Reads mapped to the final assembled genome.

## Data Availability

The data that support the findings of this study are available from the corresponding author upon reasonable request. Genome sequences are available in GenBank under accession numbers indicated in the Supplemental Material.

## Supplementary Materials

The following supporting information can be downloaded at: https://www.mdpi.com/article/10.3390/v18060629/s1, Table S1: Accession numbers of the sequences used in this study; Table S2: Putative SNPs and INDELs in CDS regions unique to the CTGV isolates described in this study; Figure S1: Detailed visualization of the CTGV Subtree; Figure S2: Phenotype of viral plaques produced by the CTGV isolates sequenced in this study; Figure S3: Area of viral plaques produced by the CTGV isolates in HeLa cells.

## Abbreviations

The following abbreviations are used in this manuscript:

VACV	Vaccinia virus
CTGV	VACV Cantagalo strain
OPXV	Orthopoxvirus
PPXV	Parapoxvirus
Ct	Cycle Threshold
PCR	Polymerase Chain reaction
SNP	Single Nucleotide Polymorphism
INDEL	Insertion and Deletion
PopART	Population Analysis with Reticulate Trees
MJN	Median Joining Network
GO	State of Goiás
PE	State of Pernambuco
BA	State of Bahia
SP	State of São Paulo
ITR	Inverted Terminal Repeat
