# Supplementary material for: Genomic Insights into the Spread of Vaccinia Virus Strain Cantagalo to Rural Regions of Northeastern Brazil
Source: Viruses. 2026 May 30;18(6):629. doi: 10.3390/v18060629 (PMC13307827; doi:10.3390/v18060629)
Supplement: Supplementary file 1 [file viruses-18-00629-s001.zip › Table S2.pdf]

Table S2. Putative SNPs and INDELs in CDS regions unique to the CTGV isolates described in this study.

Clinical isolate	Genome position (pb)	Type of alteration	Effect	VACV-Cop ortholog [b]	Function
IBI-05	2,587	SNP (G → A)	silent	VACV WR-002|217	TNF-alpha-receptor-like protein
IBI-05	5,013	SNP (C → T)	silent	C12L	Serine protease inhibitor 1
IBI-05	10,259	SNP (C → T)	silent	VACV WR-015	Ankyrin-like 77 kDa cowpox host-range protein
IBI-05	12,084	SNP (C → T)	Q540K	C9L	Ankyrin-like protein, antagonizes IRF-I
BC-04	13,744	SNP (C → G)	C19S	C8L	Hypothetical protein
BC-04	15,859	SNP (G → A)	silent	C4L	NFkB inhibitor
TE-09	16,151	SNP (C → T)	silent	C4L	NFkB inhibitor
BC-02	16,383	SNP (C → T)	silent	C4L	NFkB inhibitor
LO-01	16,043	SNP (C → T)	silent	C4L	NFkB inhibitor
BC-04	17,875	SNP (G → A)	H84Y	C2L	BTB-Kelch protein, NFkB inhibitor
BC-04	18,115	Deletion (2 nt)	FS/SC [a]	C2L	BTB-Kelch protein, NFkB inhibitor
BC-04	18,836	Deletion (1 nt)	FS/SC	C2L	BTB-Kelch protein, NFkB inhibitor
BC-04	19,551	SNP (C → T)	R125K	C1L	Putative Bcl-2 protein, host defense modulator
TE-09	20,125	SNP (C → T)	G84S	N1L	Putative Bcl-2 protein, NFkB inhibitor
BC-02	12,359	SNP (C → T)	G84S	N1L	Putative Bcl-2 protein, NFkB inhibitor
TE-09	20,847	SNP (C → T)	R54K	N2L	Putative Bcl-2 protein, alpha-amanitin target; virulence factor
BC-02	21,079	SNP (C → T)	R54K	N2L	Putative Bcl-2 protein, alpha-amanitin target; virulence factor
LO-01	20,739	SNP (C → T)	R54K	N2L	Putative Bcl-2 protein, alpha-amanitin target; virulence factor
BC-02	21,197	SNP (C → T)	E15K	N2L	Putative Bcl-2 protein, alpha-amanitin target; virulence factor
BC-04	22,244	SNP (A → G)	F36L	M1L	Ankyrin-like protein
IBI-05	23,263	SNP (C → T)	E77K	M2L	NFkB inhibitor
IBI-05	23,452	SNP (G → A)	L14F	M2L	NFkB inhibitor
TE-09	23,325	SNP (C → T)	D256N	K1L	Ankyrin-like protein, host-range; antagonizes IRF-I
TE-09	23,817	SNP (C → T)	D92N	K1L	Ankyrin-like protein, host-range; antagonizes IRF-I
IBI-05	25,978	SNP (C → T)	silent	K3L	eIF2 alpha-like PKR inhibitor
IBI-05	27,670	Insertion (G)	FS/SC	K5L	Putative monoglyceride lipase
TE-09	27,455	SNP (G → A)	P48L	K5L	Putative monoglyceride lipase

BC-04	27,945	SNP (C → T)	silent	K7R	NFkB inhibitor; inhibits IRF-3 activation and IFN- I expression
IBI-05	36,296	SNP (C → T)	E10K	F10L	Serine-threonine kinase 2
TE-09	37,824	SNP (C → T)	G383E	F12L	KLC-like protein, WV formation associated protein
TE-09	46,725	SNP (C → T)	silent	E4L	DNA-dependent RNA polymerase 30 subunit
TE-09	48,340	SNP (G → A)	silent	E5R	Virosome component protein
BC-02	48,572	SNP (G → A)	silent	E5R	Virosome component protein
LO-01	48,232	SNP (G → A)	silent	E5R	Virosome component protein
IBI-05	49,308	SNP (G → A)	D116N	E6R	Core protein, virus morphogenesis protein
BC-04	49,817	SNP (G → A)	R449K	E6R	Core protein, virus morphogenesis protein
IBI-05	50,607	SNP (C → T)	H549Y	E6R	Core protein, virus morphogenesis protein
BC-04	50,941	SNP (C → T)	silent	E8R	Core protein, substrate for F10 kinase
TE-09	51,197	SNP (C → T)	silent	E8R	Core protein, substrate for F10 kinase
BC-02	51,429	SNP (C → T)	silent	E8R	Core protein, substrate for F10 kinase
LO-01	51,089	SNP (C → T)	silent	E8R	Core protein, substrate for F10 kinase
BC-04	52,969	SNP (C → T)	silent	E9L	DNA polymerase
TE-09	58,486	SNP (G → A)	silent	O1L	Extracellular signal-regulated kinase-activating protein
BC-02	56,718	SNP (G → A)	silent	O1L	Extracellular signal-regulated kinase-activating protein
BC-04	57,928	SNP (G → A)	S308L	I1L	Telomere-binding protein
BC-02	68,831	SNP (G → A)	L101F	G1L	Insulin metalloproteinase-like protein
BC-04	76,146	SNP (C → T)	G337S	L3L	Internal virion protein, required for early transcription
IBI-05	79,556	SNP (G → A)	G101E	J2R	Thymidine kinase
TE-09	81,450	SNP (C → T)	silent	J5L	Integral component of virus entry|fusion complex protein
BC-02	81,682	SNP (C → T)	silent	J5L	Integral component of virus entry|fusion complex protein
BC-04	83,320	SNP (G → A)	G616R	J6R	DNA-dependent RNA polymerase subunit RPO147
BC-04	86,123	SNP (G → A)	R88K	H2R	Integral component of virus entry|fusion complex protein
BC-04	87,516	SNP (C → T)	silent	H4L	RNA polymerase-associated protein RAP94
IBI-05	92,786	SNP (G → A)	silent	D1R	mRNA capping enzyme large subunit

IBI-05	93,288	SNP (C → T)	silent	D1R	mRNA capping enzyme large subunit
IBI-05	99,782	SNP (C → T)	silent	D6R	Early gene transcription factor VETF 70 kDa small subunit
TE-09	99,496	SNP (G → A)	silent	D6R	Early gene transcription factor VETF 70 kDa small subunit
BC-02	99,728	SNP (G → A)	silent	D6R	Early gene transcription factor VETF 70 kDa small subunit
LO-01	99,385	SNP (G → A)	silent	D6R	Early gene transcription factor VETF 70 kDa small subunit
IBI-05	100,851	SNP (C → T)	S531F	D6R	Early gene transcription factor VETF 70 kDa small subunit
BC-04	100,740	SNP (C → T)	silent	D7R	DNA-dependent RNA polymerase subunit RPO18
BC-04	103,515	SNP (G → A)	silent	D11L	Nucleoside triphosphate phosphohydrolase-I, NPH-I; transcription elongation
LO-01	104,758	SNP (G → A)	S210L	D11L	Nucleoside triphosphate phosphohydrolase-I, NPH-I; transcription elongation
BC-04	111,218	SNP (A → G)	silent	A3L	Major core protein 4b precursor
IBI-05	112,010	SNP (G → A)	S225L	A4L	39 kDa core protein
BC-04	113,032	SNP (C → T)	silent	A6L	Core protein, required for virus envelope biogenesis
BC-04	113,045	SNP (C → T)	R270K	A6L	Core protein, required for virus envelope biogenesis
BC-04	114,237	SNP (G → A)	silent	A7L	Early gene transcription factor VETF 82 kDa
IBI-05	121,078	SNP (G → A)	silent	A11R	Viral membrane biogenesis protein
IBI-05	122,458	SNP (G → A)	silent	A14L	Phosphorylated MV membrane protein
IBI-05	124,107	SNP (T → C)	I12V	A16L	Integral component of virus entry|fusion complex protein
BC-04	125,608	SNP (G → A)	M439I	A18R	DNA helicase
BC-04	127,803	SNP (G → A)	R74K	A22R	Holliday junction resolvase
BC-04	127,853	SNP (C → T)	H91Y	A22R	Holliday junction resolvase
BC-04	130,088	SNP (G → A)	A271T	A24R	DNA-dependent RNA polymerase subunit RPO132
LO-01	134,133	SNP (C → A)	R53I	A25L	Cowpox A-type inclusion protein
BC-04	134,206	SNP (C → T)	D36N	A25L	Cowpox A-type inclusion protein
BC-04	135,359	SNP (G → T)	H364N	A25L	Cowpox A-type inclusion protein
BC-04	135,392	SNP (C → T)	G353R	A25L	Cowpox A-type inclusion protein
IBI-05	136,566	SNP (C → T)	E120V	A26L	Cowpox A-type inclusion protein, binds to laminin

BC-04	136,829	SNP (G → A)	P390L	A26L	Cowpox A-type inclusion protein, binds to laminin
TE-09	137,493	SNP (C → A)	V198L	A26L	Cowpox A-type inclusion protein, binds to laminin
BC-04	137,555	SNP (C →T)	S178N	A26L	Cowpox A-type inclusion protein, binds to laminin
IBI-05	142,090	SNP (G → A)	silent	A33R	EV envelope phosphoglycoprotein
IBI-05	143,447	SNP (G → A)	G162R	A35R	MHC-II antigen presentation inhibitor
BC-04	144,576	SNP (G → A)	M261I	A37R	Hypothetical protein
BC-02	145,112	SNP (G → A)	I35K	VACV-WR-161	Hypothetical protein
BC-04	144,834	SNP (C →T)	P49L	VACV-WR-161	Hypothetical protein
IBI-05	150,037	SNP (C → T)	silent	270	Hypothetical protein
BC-04	151,884	SNP (G → A)	R236K	A46R	NFkB inhibitor; Toll|IL1-receptor-like protein
IBI-05	153,055	SNP (G → A)	silent	A47L	Immunoprevalent protein
BC-04	153,953	SNP (G → A)	silent	A49R	NFkB inhibitor; putative phosphotransferase
IBI-05	159,352	SNP (C → T)	P185L	A55R	Kelch-like protein
IBI-05	160,347	SNP (G → A)	D517N	A55R	Kelch-like protein
BC-04	160,599	SNP (G → A)	E179K	A56R	Hemagglutinin
TE-09	169,023	SNP (G → A)	D177N	B8R	Soluble interferon-II receptor-like protein
BC-02	169,255	SNP (G → A)	D177N	B8R	Soluble interferon-II receptor-like protein
LO-01	168,915	SNP (G → A)	D177N	B8R	Soluble interferon-II receptor-like protein
IBI-05	169,995	SNP (G → A)	G4E	B10R	Kelch-like protein
BC-04	169,983	SNP (G → A)	silent	B10R	Kelch-like protein
IBI-05	170,165	SNP (G → A)	E61K	B10R	Kelch-like protein
LO-01	170,545	SNP (G → A)	silent	B12R	Serine|threonine protein kinase
TE-09	170,653	SNP (G → A)	silent	B12R	Serine|threonine protein kinase
BC-02	170,885	SNP (G → A)	silent	B12R	Serine|threonine protein kinase
IBI-05	171,906	SNP (G → A)	E37K	B13R	Serine protease inhibitor 2
BC-04	172,032	SNP (C → T)	silent	B13R	Serine protease inhibitor 2
IBI-05	175,373	SNP (C → A)	R24I	B17R	Hypothetical protein
BC-04	175,699	SNP (C → A)	silent	B18R	Ankyrin-like protein
BC-04	176,521	SNP (C → T)	silent	B18R	Ankyrin-like protein
IBI-05	177,815	SNP (G → A)	R148K	B19R	IFN-I-receptor-like secreted glycoprotein
IBI-05	182,161	SNP (G → A)	silent	C12L	Serine protease inhibitor 1
IBI-05	184,587	SNP (C → T)	silent	VACV WR-002|217	TNF-alpha-receptor-like protein

[a]FS/SC: frameshift mutation with the introduction of a downstream stop codon.
[b] When there is no ortholog in VACV-Cop genome, the ortholog in VACV-WR is indicated.
CTGV isolates are indicated by different colors. Putative SNPs and INDELs were detected when aligning the isolate genome sequences against a 50% consensus sequence of the 18 CTGV genomes sequenced to date. Putative SNPs and INDELs were confirmed by mapping the reads to the assembled genome. Nt: nucleotide.
