# Supplementary material for: Genomic Insights into the Spread of Vaccinia Virus Strain Cantagalo to Rural Regions of Northeastern Brazil
Source: Viruses. 2026 May 30;18(6):629. doi: 10.3390/v18060629 (PMC13307827; doi:10.3390/v18060629)
Supplement: Supplementary file 1 [file viruses-18-00629-s001.zip › Figure S1.pdf]

Figure S1: Detailed visualization of the CTGV subtree.

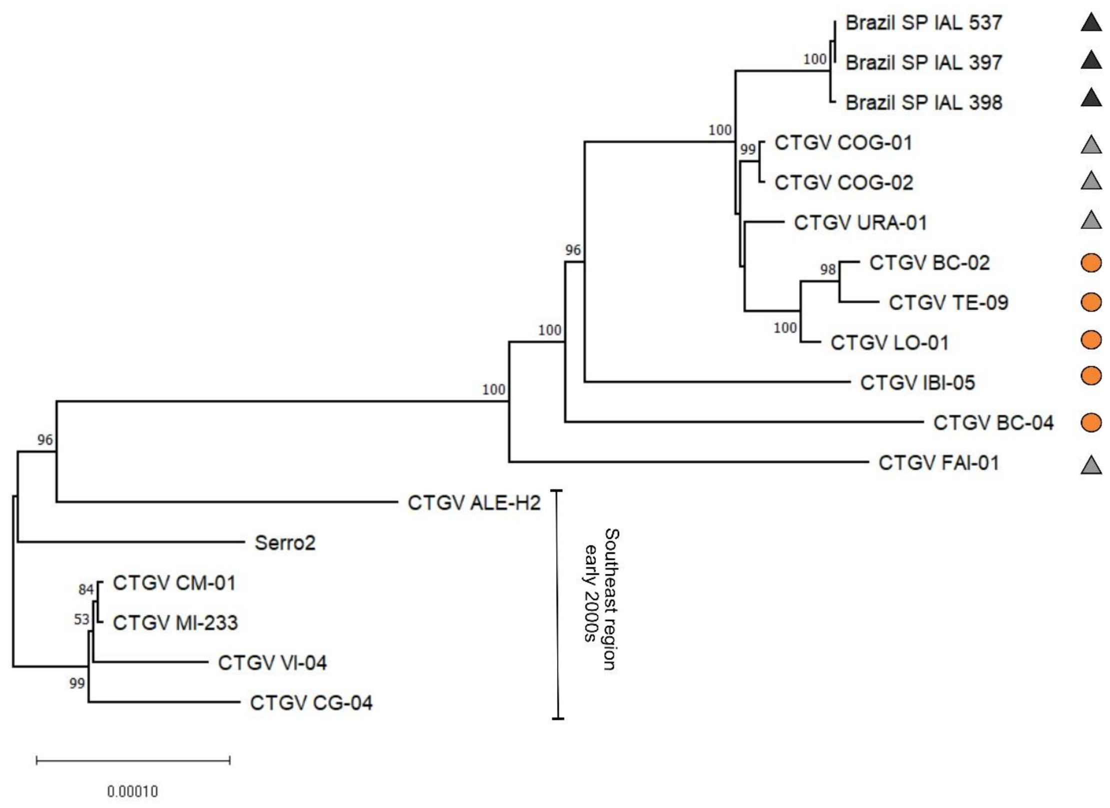

The CTGV subtree was extracted from the phylogenetic tree shown in Figure 2A using MEGA 11 and presented with extended branch lengths and increased taxon separation. Maximum likelihood phylogenetic tree, opting for Kimura-3-p model with empirical base frequencies, 3-category free rate model, and 1,000 bootstraps. Orange circles: CTGV isolates from Pernambuco (BC-02, BC-04, TE-09, and LO-01) and Bahia (IBI-05) sequenced in this work. Gray and black triangles: CTGV genomes from Goiás, 2022 and São Paulo, 2023, respectively. Bootstrap values >50% are shown next to branch nodes. The scale bar indicates the number of substitutions per site.
