# Supplementary material for: Genomic Insights into the Spread of Vaccinia Virus Strain Cantagalo to Rural Regions of Northeastern Brazil
Source: Viruses. 2026 May 30;18(6):629. doi: 10.3390/v18060629 (PMC13307827; doi:10.3390/v18060629)
Supplement: Supplementary file 1 [file viruses-18-00629-s001.zip › Figure S2.pdf]

Figure S2: Phenotype of viral plaques produced by the CTGV isolates sequenced in this study.

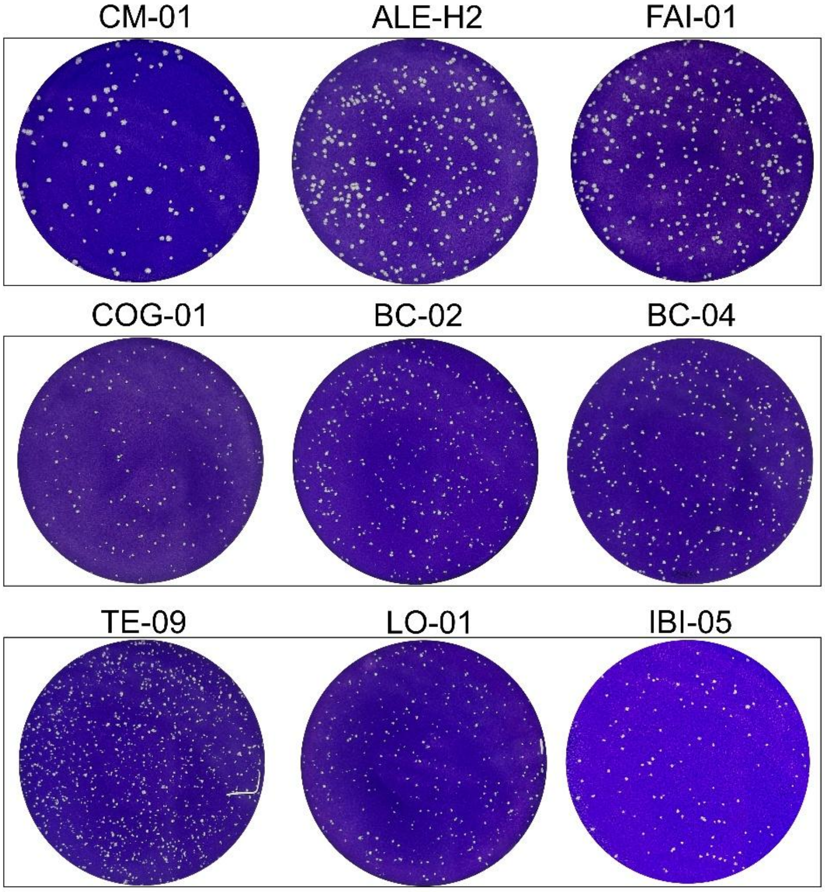

BSC-40 cells infected with the indicated CTGV isolates for 48 hours were fixed and stained with 10% formaldehyde/0.1% crystal violet. Representative wells were photographed. CM-01: Isolate from Rio de Janeiro, 2000; ALE-H2: Isolate from Espírito Santo, 2006; FAI-01 and COG-01: Isolates from Goiás, 2022; BC-02, BC-04, TE-09, and LO-01: Isolates from Pernambuco, this study; IBI-05: Isolate from Bahia, this study.
