# Supplementary material for: Genomic Insights into the Spread of Vaccinia Virus Strain Cantagalo to Rural Regions of Northeastern Brazil
Source: Viruses. 2026 May 30;18(6):629. doi: 10.3390/v18060629 (PMC13307827; doi:10.3390/v18060629)
Supplement: Supplementary file 1 [file viruses-18-00629-s001.zip › Figure S3.pdf]

Figure S3: Area of viral plaques produced by the CTGV isolates in HeLa cells.

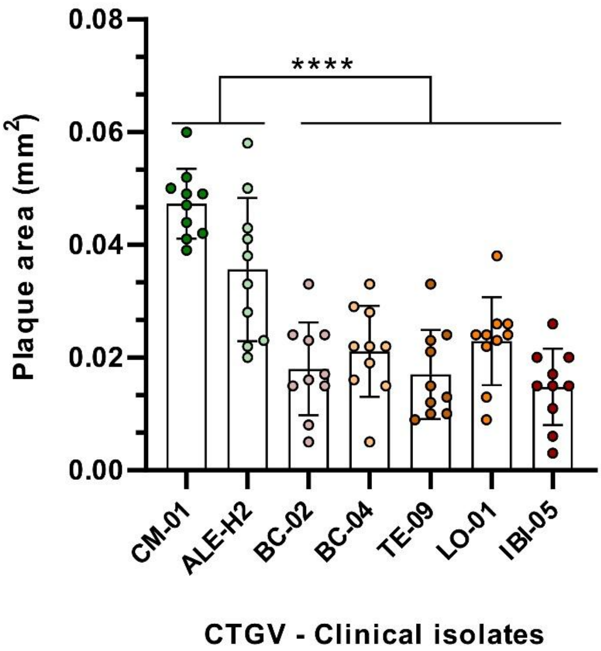

HeLa cells were infected with the indicated CTGV isolates for 48 hours, fixed, and stained with 10% formaldehyde/0.1% crystal violet. Random plaques were photographed and the area was measured. CM-01: Isolate from Rio de Janeiro, 2000; ALE-H2: Isolate from Espírito Santo, 2006; BC-02, BC-04, TE-09, and LO-01: Isolates from Pernambuco, this study; IBI-05: Isolate from Bahia, this study. Mean ± SD; asterisk: $p<0.0001$ (One-way ANOVA followed by Bartlett's test).
