# Supplementary material for: Genomic Insights into the Spread of Vaccinia Virus Strain Cantagalo to Rural Regions of Northeastern Brazil
Source: Viruses. 2026 May 30;18(6):629. doi: 10.3390/v18060629 (PMC13307827; doi:10.3390/v18060629)
Supplement: Supplementary file 1 [file viruses-18-00629-s001.zip › Table S1.pdf]

Table S1. Accession numbers of the sequences used in this study.

Clinical isolate	Accession number *
HPXV VK05	BK013341
HPXV VK08	BK013342
HPXV MNR-76	DQ792504
VARV Bgd75	DQ437581
VARV Brazil 1966	DQ441419
VACV Serro2	KF179385
VACV Brazil SP IAL398	OR515686
VACV Brazil SP IAL537	OR515687
VACV Brazil SP IAL397	OR515685
VACV TianTan TP3	KC207810
VACV TianTan TT5	KC207811
VACV TianTan TT8	JX489135
VACV Lister	DQ121394
VACV Copenhagen	M35027
VACV LC16m8	AY678275
VACV IHDW1	KJ125439
VACV DUKE	DQ439815
VACV 3737	DQ377945
VACV Tashkent-TKT3	KM044309
VACV Tashkent-TKT4	KM044310
VACV WR	AY243312
VACV IOC B388	KT184691
VACV IOC B141	KT184690
VACV DPP10	JN654977
VACV DPP11	JN654978
VACV DPP13	JN654980
VACV DPP15	JN654981
VACV DPP17	JN654983
VACV DPP20	JN654985
VACV Wyeth A111	OP751801
VACV ACAM2000	AY313847
Mulford 1902	MF477237
CTGV CM-01	KT013210

CTGV MI-233	MW018153
CTGV VI-04	MW018154
CTGV CG-04	MW018155
CTGV ALE-H2	MW018156
CTGV URA-01	OQ754427
CTGV FAI-01	OQ754424
CTGV COG-01	OQ754425
CTGV COG-02	OQ754426
CTGV BC-02	PX423480
CTGV BC-04	PX423481
CTGV LO-01	PX423482
CTGV TE-09	PX423483
CTGV IBI-05	PX423484
CPXV GRI-90	X94355
CPXV Finland 2000	HQ420893
CPXV Brighton Red	AF482758
CPXV Ger90	HQ420896
CPXV Ger2010 MKY	LT896721
CPXV HumBer 07/1	KC813509
CPXV Ger/2013 Alpaca	LT896719
CPXV No-H2	OM460002
CPXV HumAac09/1	KC813508
CPXV RatGer09/1	KC813503
CMPV	NC003391
TATPV	DQ437594
MPXV Brazil BA	EPI ISL 14995619
MPXV USA 2003 044	DQ011153
MPXV DRC CRSN-1 2023	EPI ISL 19004044
MPXV Congo 8	KJ642613

* GenBank accession numbers, except EPI ISL 14995619 and EPI ISL 19004044, which are GISAID Accession numbers.

VACV, vaccinia virus, HPXV, horsepox virus, VARV, variola virus, CTGV, vaccinia virus strain Cantagalo, CPXV, cowpox virus, CMPV, camelpox virus, TATPV, taterapox virus, MPXV, monkeypox virus.
